# Detection of *Candida albicans* biofilm proteins induced by glucose, lactose, soy protein, and iron

**DOI:** 10.4317/jced.55787

**Published:** 2019-06-01

**Authors:** Indah Listiana-Kriswandini, Markus Budi-Rahardjo, Pratiwi Soesilawati, Aileen Prisca-Suciadi

**Affiliations:** 1 DDS, MSc, PhD. Department of Oral Biology, Faculty of Dental Medicine, Universitas Airlangga, Surabaya, Indonesia; 2 DDS, MSc. Department of Oral Biology, Faculty of Dental Medicine, Universitas Airlangga Surabaya, Indonesia; 3 DDS, MSc, PhD. Department of Oral Biology, Faculty of Dental Medicine, Universitas Airlangga Surabaya, Indonesia; 4 Undergraduate student, Faculty of Dental Medicine, Universitas Airlangga, Surabaya, Indonesia

## Abstract

**Background:**

Oral candidiasis is one of the most common fungal infections, which attack the mucosa of the oral cavity. These lesions are mostly caused by the fungal species *Candida albicans*. *Candida albicans* is included in the normal oral microorganisms that are opportunistic pathogens, and its presence is quite large, which can reach 75% of the total oral fungal population. Research on specific proteins of Candida biofilm can be an alternative to early prevention of oral infections such as Oral Candidiasis. This biofilm protein can be used as a reference in making kits to detect the presence of microbes that cause infectious diseases. The purpose of this study was to determine molecular weight of *Candida albicans* biofilm protein induced by 5% glucose, 5% lactose, soy protein, and 5% iron.

**Material and Methods:**

This experimental laboratory study used SDS-PAGE electrophoresis to determine the molecular weight of *Candida albicans* biofilm proteins induced by glucose 5%, lactose 5%, soy protein, and iron 5%.

**Results:**

Biofilm induced by 5% glucose shows four protein bands: 71,6 kDa; 56,1 kDa; 49,7 kDa; and 41 kDa. Biofilm induced by 5% lactose shows seven protein bands: 71 kDa; 61,2 kDa; 57,7 kDa; 55,3 kDa; 48,9 kDa; 39,5 kDa; and 29,8 kDa. Biofilm induced by soy protein shows one protein band: 49,4 kDa. Biofilm induced by 5% iron shows one protein band: 51,1 kDa.

**Conclusions:**

*Candida albicans* biofilm induced by 5% glucose has four protein band candidates, 5% lactose has seven candidates of protein band, and soy protein and 5% iron each has a candidate of protein band, which can be used as a target for the detection of oral Candidiasis.

** Key words:**Biofilm protein, Candida albicans, molecular weight, oral candidiasis.

## Introduction

Oral candidiasis is one of the most common fungal infections, which attack the mucosa of the oral cavity. These lesions are mostly caused by the fungal species *Candida albicans* (1,2). *Candida albicans* is included in the normal oral microorganisms that are opportunistic pathogens, and its presence is quite large, which can reach 75% of the total oral fungal population (2).

Microorganisms in the oral cavity, including *Candida albicans* species, live by colonizing until they form a biofilm. Biofilm will then develop to form a microenvironment for microorganisms with the aim of maintaining their lives (3). Aside from being a form of survival, biofilms also function as an adherence to microorganisms to invade the tissue in the host’s body. The local effects of biofilms on the host’s body tissues are very complex, can be beneficial or harmful, and these effects can change over time. However, the National Institutes of Health estimates that biofilms play an important role in 80% of microbial infections in the United States (4).

Carbohydrates, proteins and iron are essential substances needed by the body to stay alive and develop, so that the substance is almost always present in food ingredients consumed by humans. These substances not only affect the growth of the host body, but also the growth of microorganisms that live in it. Carbohydrates, proteins and iron can each affect the expression of biofilm microorganisms differently, resulting in different virulence effects (5).

To conduct appropriate therapy for oral infections, in this case Oral Candidiasis, not only knowledge is needed about how severe the infection is, but it is also necessary to know the cause. Therefore, we need a device that can detect different expressions of each type of microorganism biofilm.

In the manufacture of disease detection devices, a biomarker is needed. Biomarkers are an indication or objective indication of medical conditions observed from outside the patient, which can be measured accurately (6). To obtain an accurate biomarker, it is necessary to first find the molecular weight and expression strength of each specific protein that arises from the microorganisms concerned, in this case the *Candida albicans* biofilm induced by glucose, lactose, soy protein, and iron (FeCl2).

Based on this, the authors examined the effect of exposure to glucose, lactose, protein, and iron on the expression of specific proteins of *Candida albicans* biofilm. The purpose of this study was to determine each specific protein profile of *Candida albicans* biofilm which had been exposed by glucose, lactose, protein and iron.

## Material and Methods

This research was conducted in Microbiology Laboratory and Biomedical Laboratory, Faculty of Medical, Brawijaya University, Malang. Procedures for detecting the biofilm proteins include: microbes culture, biofilm growth, isolation of biofilm proteins, Sodium Dodecyl Sulfate (SDS) Poly Acrilamide Gel Electrophoresis (PAGE) electrophoresis, and analyze the molecular weight using Gel Doc ™ EZ Imager software (BIO RAD).

*Candida albicans* were cultured and replicated in Saboraud Dextrose Agar (SDA) medium (OXOID) and ensuring fungal growth occurs with an indication of turbidity equivalent to the Mc Farland 8 standard, then it was observed in a microscope (Olymphus) to ensure the results of the culture are not contaminated. On biofilm growth, *Candida albicans* was inserted in a 50 ml Saboraud Dextrose Broth (SDB)(OXOID) tube supplemented with 5% glucose (SIGMA), 5% lactose (SIGMA), 5% Iron (FeCl2) (Choice Chem Ltd.) on each tube, and *Candida albicans* inserted in a 50 ml SDB tube (as a control group). For soy protein, *Candida albicans* was inserted in a 50 ml tube with Trypticase Soy Broth (TSB)(OXOID) supplemented with 1% glucose, then incubated overnight at 37°C to grow *Candida albicans* biofilm (7).

Isolation of biofilm protein was done by scraping biofilms formed on each base of the Erlenmeyer tube by adding PBS + Tween 0.05% and being transferred to Eppendorf. Centrifuge at 12.000 rpm x 10 minutes. Transfer the supernatant to Eppendorf and precipitated it with 1: 1 alcohol, then incubate one night. Calculates protein concentration using Nanodrop (7).

The method of SDS-PAGE electrophoresis begins with preparation of the gel first. After the gel is formed with a mixture of separating gel 12% and 4% stacking gel, the plate was mounted on an electrophoresis device, then the buffer was poured on the electrophoresis vessel. Then injection of samples on the gel that has been made by inserting 10 µl of isolated protein sample in each *Candida albicans* biofilm in TSB medium, glucose induced *Candida albicans* biofilm on SDB media, lactose induced *Candida albicans* biofilm on SDB media, and *Candida albicans* biofilm on SDB media of iron induced (FeCl2) was added with 10µl Tris-Cl + 20µl Reducing Sample Buffer (RSB)(NOVEX), and put into the micro tube, then heated in a water heater at 100oC for 5 minutes. After being cooled, the sample is inserted in gel wells with a volume of 20 µl for each well. The power supply for electrophoresis is turned on with an electric current of 30 mA and 100 V. After the gel removed from the plate, staining and washing the gel is carried out. Staining is done by soaking the gel in a staining solution for ± 4 hours – overnight in shaker incubator (8).

The SDS-PAGE electrophoresis gel was documented in the form of images. The results in the form of this image are analyzed for measuring protein molecular weight of the protein bands using Gel Doc ™ EZ Imager software. The program reads the molecular weight of the protein bands that appear in each column.

## Results

**Figure 1:**
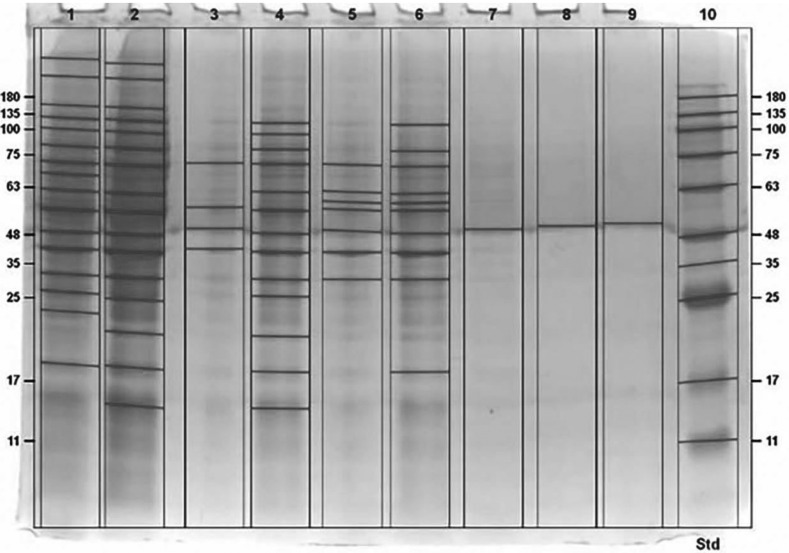
The result of SDS-PAGE electrophoresis. KDa = molecular weight in units of kilo dalton, lane 1 = standard (planktonic), lane 2 = glucose-induced whole cell, lane 3 = glucose-induced biofilm, lane 4 = lactose-induced whole cell, lane 5 = lactose-induced biofilm, lane 6 = soy protein-induced whole cell, lane 7 = soy protein-induced biofilm, lane 8 = iron-induced whole cell, lane 9 = iron-induced biofilm, lane 10 = marker.

After the SDS-PAGE electrophoresis procedure with 4% stacking gel and 12% separating gel completed, the gel was removed and then stained using commasie blue so that the image of protein bands could appear. Then the gel was scanned to read the molecular weight of the protein bands using Gel Doc ™ EZ Imager software. The protein marker is Jena Bioscience Blueray, whereas this marker protein contains 10 standards in the range 11-180 kDa. By using the Gel Doc ™ EZ Imager software, we can determine the molecular weight protein with kDa (kiloDalton) unit (Fig. 1).

From analysis using EZ Gel Doc ™ Imager software, there are four protein bands appeared on glucose-induced biofilms, seven protein bands appeared on lactose-induced biofilms, one protein band on soy protein-induced biofilms, and one protein band on iron-induced biofilm with each molecular weight as shown in  Table 1, which each band can be said as the candidate in determining biofilm specific proteins from each inducer.

**Table 1:**
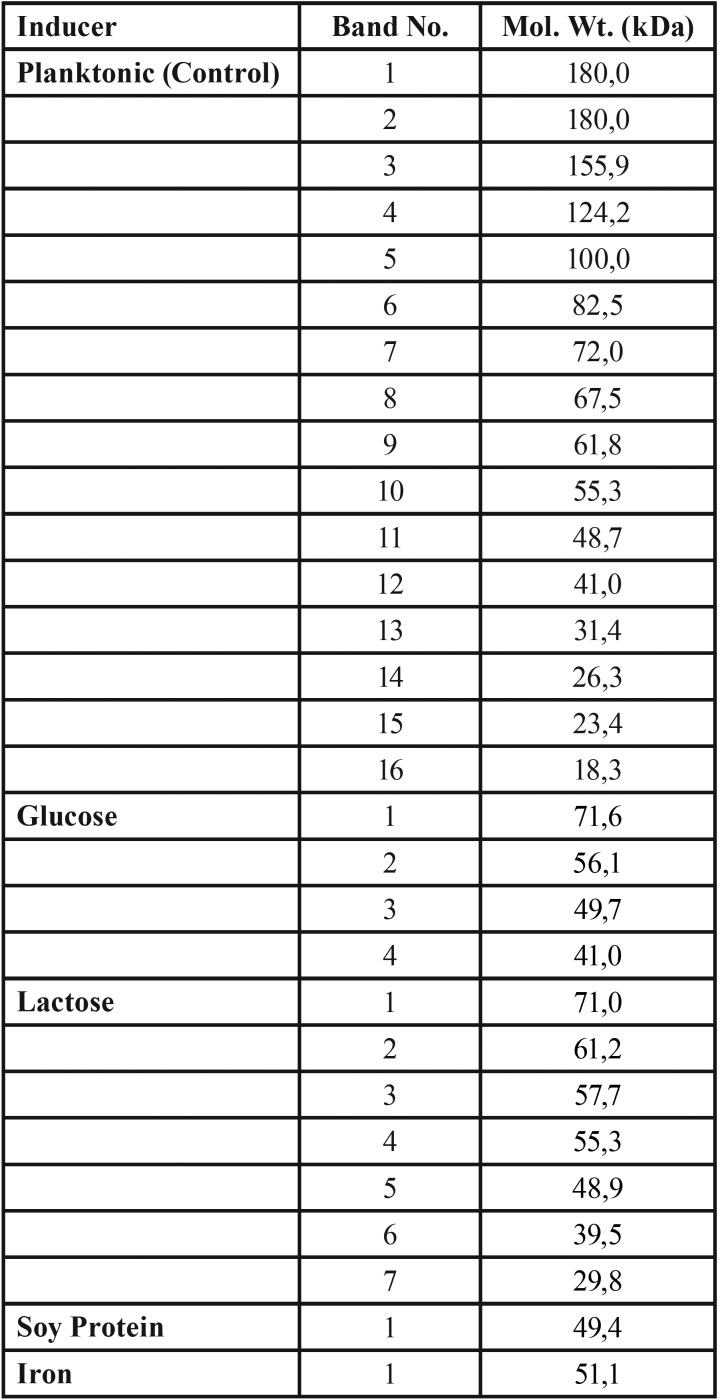
The molecular weight of each induced protein biofilm.

## Discussion

This study uses several inducers which will be induced in *Candida albicans*, there are 5% glucose, 5% lactose, soy protein, and iron (FeCl2) 5%. The inducer is used as a trigger for the formation of *Candida albicans* biofilms which are expected to produce different protein band expressions. This 5% concentration was chosen to see its effect on the biofilm growth rate of *Candida albicans*. But in the results of this study, the concentration does not always give a positive result for the biofilm growth of *Candida albicans*, iron-induced *Candida albicans* producing only a small amount of protein, so there is almost no protein band appeared in after the electrophoresis. This is estimated because iron with a concentration of 5% is probably too large to be induced to *Candida albicans*. According to Mishra *et al.* (9), the condition of excess iron in Candida can increase the methylation of DNA, which in turn can cause the activity of the iron receiving gene to be depressed. In addition, according to Lan *et al.* (5), the condition of iron overload can suppress the expression of the Sfu1 gene and inhibit Sef1 expression so that it also suppresses the iron receiving gene. Therefore, the concentration of iron-induced Candida biofilm protein is obtained in only small amount.

The SDS-PAGE electrophoresis results in the 5% glucose induced *Candida albicans* treatment found that the pellet protein consisted of 16 protein bands in the range from 14 kDa to 180 kDa. While biofilm protein showed 4 protein bands with different molecular weights than planktonic, namely 71.6 kDa, 56.1 kDa, 49.7 kDa, and 41 kDa. Although some pellet proteins and biofilms that appear have different molecular weights than their planktonic, the location of these proteins is still on one line with planktonic proteins. So that the protein that appears is estimated to be the same type of protein as planktonic. The four proteins that appear are also thought to be specific protein candidates that have a role in *Candida albicans* biofilm when induced by 5% glucose. To find out more precisely whether the four proteins actually have a specific role in glucose-induced *Candida albicans* biofilm, further research needs to be done by tracking the amino acid sequence.

Vediyappan *et al.* (10), in their study succeeded in identifying several *Candida albicans* biofilm proteins. Based on the study, biofilm protein *Candida albicans* at 71.6 kDa was estimated as heat shock protein 70, 49.7 kDa was estimated as NADP-specific glutamate dehydrogenase protein, and 41 kDa was estimated as an adenosine kinase protein.

Among the identified proteins, there are proteins with a type of heat shock protein. Heat shock protein is a protein formed in response to thermal stress. In *Candida albicans*, heat shock protein has a role in controlling basic physiological activity and virulence through its interaction with various kinds of regulators from the cellular signaling pathway. Thus, heat shock proteins can induce resistance of *Candida albicans* to anti-fungal drugs (11). Therefore, to anticipate this, further research is needed to disrupt the normal function of heat shock proteins to be able to inhibit the growth and resistance of *Candida albicans*.

The SDS-PAGE results in 5% lactose induced *Candida albicans* found that the pellet protein consisted of 13 protein bands ranging from 13.4 kDa to 112.4 kDa. While the biofilm protein showed 6 protein bands which had different molecular weights than planktonic, namely 71 kDa, 61.2 kDa, 55.3 kDa, 48.9 kDa, 39.5 kDa, and 29.8 kDa. This biofilm protein also produces 1 different protein band, which is not present in planktonic, that is 57.7 kDa. The seven proteins are thought to be the candidate of specific protein of 5% lactose-induced *Candida albicans*, but it is necessary to trace the amino acid sequence to find out whether one different emerging protein plays a specific role in lactose-induced *Candida albicans* biofilm.

Referring to the previous research reference (11), protein bands with a molecular weight of 71 kDa are estimated as heat shock protein 70, 61.2 kDa is estimated as heat shock protein 60, 48.9 kDa is estimated as mannose-6-phosphate isomerase, 39.5 kDa as adenosine kinase, and 29.8 kDa as hypothetical CaO19 protein. While the additional protein at 57.7 kDa is estimated as a putative glucose-6-phosphate dehydrogenase protein.

As for glucose induction, lactose induction in *Candida albicans* can also initiate the formation of heat shock protein. In a previous study, it was revealed that heat shock protein can increase alternating activity from yeast cells to hyphae, thereby also increasing *Candida albicans* biofilm formation (12). So it can be predicted that with glucose and lactose induced, virulence and biofilm growth can increase.

An additional protein band that appears in lactose induction is thought to be identified as the putative glucose-6-phosphate dehydrogenase protein. The protein in the biological process of *Candida albicans* plays a role in the process of glucose metabolism, namely by catalyzing the oxidative pentose-phosphate pathway which provides a carbohydrate catabolism pathway besides glycolysis. Thus the protein can increase glucose breakdown in *Candida albicans* and increase its biofilm growth.

The SDS-PAGE results in the treatment of *Candida albicans* induced by soy protein and iron found that each biofilm protein only showed one protein band that had a different molecular weight than its planktonic, that is 49.4 kDa in induction of soy protein, and 51.1 kDa for iron induced. These proteins have a different molecular weight than the planktonic, but are still on the same line (approximately 1 kDa). Because only one protein band is expressed, the protein is thought to be a specific protein that plays a role in *Candida albicans* biofilm induced by soy protein and iron. But it still needs to be traced to the amino acid sequence to find out the truth.

Referring to the previous study, both of these proteins were estimated as NADP-specific glutamate dehydrogenase proteins (5). The protein in *Candida albicans* acts as a catalyst in oxydoreductase activity, namely oxidation and reduction of hydrogen components in cells. With the presence of these catalysts can increase the respiration and metabolism of *Candida albicans*. So the induction of soy protein and iron with the right level can increase the enzyme activity and biofilm metabolism of *Candida albicans*.

Protein band with the highest density indicates that the protein has a dominant protein content of each inducer. But the protein that has the dominant content is not necessarily the most specific protein from each inducer, so that all protein bands that emerged from each inducers can be used as the candidate in determining the specific protein biofilm that can later be used as a marker in the determination of oral candidiasis due to consumption of each inducer. The names and functions of each proteins are needed further analysis to determine the function and role on the biofilm formation.
